# Characterization of a Murine Anti-laminin-1 Monoclonal Antibody (AK8) Produced by Immunization with Mouse-derived Laminin-1

**DOI:** 10.1080/17402520400014168

**Published:** 2005-03

**Authors:** Akane Kondo, Junko Inagaki, Kazuko Kobayashi, Hideo Tsukamoto, Daisuke Yamamoto, Mikiya Nakatsuka, Nobuharu Suzuki, Motoyoshi Nomizu, Satoshi Amano, Hidehiko Matsubayashi, Tatsuji Yasuda, Luis R. Lopez, Yehuda Shoenfeld, Tsunehisa Makino, Eiji Matsuura

**Affiliations:** 1 Department of Obstetrics and Gynecology Center for Specialized Clinical Science Tokai University School of Medicine Ishehara Japan; 2 Department of Cell Chemistry Okayama University Graduate School of Medicine and Dentistry Shikata-cho 2-5-1 Shikata-cho Okayama 700-8558 Japan; 3 Department of Laboratory for Molecular Science Research Tokai University School of Medicine Ishehara Japan; 4 Biomedical Computation Center Osaka Medical College Takatsuki Japan; 5 Department of Obstetrics and Gynecology Okayama University Hospital Okayama Japan; 6 Tokyo University of Pharmacy and Life Science Hachiouji Japan; 7 Shiseido Life Science Research Center Yokohama Japan; 8 Corgenix Inc Westminster Colorado USA; 9 Department of Medicine B and Center for Autoimmune Diseases Sheba Medical Center Tel-hashomer Israel

## Abstract

Laminin-1 is a structural glycoprotein that forms an integral part of the scaffolding of basement membranes, and plays an important role during embryonic development. We have recently demonstrated a significant association between anti-laminin-1 antibodies (Abs) and reproductive failure, such as recurrent spontaneous abortions and infertility-associated endometriosis in both human and mouse studies. In the present study, we established an IgM (μ,κ) monoclonal anti-laminin-1 Ab (AK8) by immunizing mice with mouse Engelbreth-Holm-Swarm sarcoma (EHS)-derived laminin-α1. The AK8 monoclonal antibody (mAb) reacted with particular peptide sequences from the globular G domain of mouse laminin-α1 chain of using ELISA and Western blot techniques. The peptide tertiary structure of the epitope recognized by AK8 mAb was predicted using eight synthesized domain peptide sequences and three consensus sequences obtained by phage displayed random peptide library. Basement membranes of endometrium of pregnant mice and humans were immunostained with AK8 mAb. Thus, AK8 mAb recognized a common structure present in the G domain of the laminin-1 chain in both mice and humans. The passive immunization of mice with AK8 mAb may represent a suitable animal model for anti-laminin-1 Ab-mediated reproductive failure.


*Clinical & Developmental Immunology*, March 2005; 12(1): 67–73

Taylor & Francis

Taylor & Francis Group

### Characterization of a murine anti-laminin-1 monoclonal antibody (AK8) produced by immunization with mouse-derived laminin-1

AKANE KONDO[1,2], JUNKO INAGAKI[2], KAZUKO KOBAYASHI[2], HIDEO TSUKAMOTO[3], DAISUKE YAMAMOTO[4], MIKIYA NAKATSUKA[5], NOBUHARU SUZUKI[6], MOTOYOSHI NOMIZU[6], SATOSHI AMANO[7], HIDEHIKO MATSUBAYASHI[1], TATSUJI YASUDA[2], LUIS R. LOPEZ[8], YEHUDA SHOENFELD[9], TSUNEHISA MAKINO[1], & EIJI MATSUURA[2]

[1]*Department of Obstetrics and Gynecology, Center for Specialized Clinical Science, Tokai University, School of Medicine, Ishehara, Japan,* [2]*Department of Cell Chemistry, Okayama University Graduate School of Medicine and Dentistry, 2-5-1 Shikata-cho, Okayama 700-8558, Japan,* [3]*Department of Laboratory for Molecular Science Research, Tokai University, School of Medicine, Ishehara, Japan,* [4]*Biomedical Computation Center, Osaka Medical College, Takatsuki, Japan,* [5]*Department of Obstetrics and Gynecology, Okayama University Hospital, Okayama, Japan,* [6]*Tokyo University of Pharmacy and Life Science, Hachiouji, Japan,* [7]*Shiseido Life Science Research Center, Yokohama, Japan,* [8]*Corgenix Inc, Westminster, Colorado, USA, and* [9]*Department of Medicine B and Center for Autoimmune Diseases, Sheba Medical Center, Tel-Hashomer, Israel*

**Abstract**
Laminin-1 is a structural glycoprotein that forms an integral part of the scaffolding of basement membranes, and plays an important role during embryonic development. We have recently demonstrated a significant association between anti-laminin-1 antibodies (Abs) and reproductive failure, such as recurrent spontaneous abortions and infertility-associated endometriosis in both human and mouse studies. In the present study, we established an IgM (μ, κ) monoclonal anti-laminin-1 Ab (AK8) by immunizing mice with mouse Engelbreth–Holm–Swarm sarcoma (EHS)-derived laminin-1. The AK8 monoclonal antibody (mAb) reacted with particular peptide sequences from the globular G domain of mouse laminin-α1 chain of using ELISA and Western blot techniques. The peptide tertiary structure of the epitope recognized by AK8 mAb was predicted using eight synthesized domain peptide sequences and three consensus sequences obtained by phage displayed random peptide library. Basement membranes of endometrium of pregnant mice and humans were immunostained with AK8 mAb. Thus, AK8 mAb recognized a common structure present in the G domain of the laminin-α1 chain in both mice and humans. The passive immunization of mice with AK8 mAb may represent a suitable animal model for anti-laminin-1 Ab-mediated reproductive failure.

**Keywords:** *Laminin-1, anti-laminin-1, monoclonal antibody, extracellular matrix protein, reproductive failure*

### Introduction

Laminins are a family of non-collagenous glycoproteins that form an integral part of the structural scaffolding of basement membranes. Laminins are large (400–900 kDa) heterotrimeric glycoproteins composed of α, β and γ chains assembled into a triple-stranded coiled-coil structure forming a cruciform structure. Five distinct α-chains, three β-chains, and three γ-chains have been identified and these subunits comprise at least 15 laminin isoforms (Tunggal et al. 2000). Laminins display tissue-specific expressions during different stages of development and are involved in diverse biological activities,

Correspondence: E. Matsuura, Department of Cell Chemistry, Okayama University Graduate School of Medicine and Dentistry, 2-5-1 Shikata-cho, Okayama 700-8558, Japan, Tel: 81 86 235 7402. Fax: 81 86 235 7404. E-mail: eijimatu@md.okayama-u.ac.jp

ISSN 1740-2522 print/ISSN 1740-2530 online © 2005 Taylor & Francis Group Ltd.
DOI: 10.1080/17402520400014168

including the promotion of cell adhesion, migration, proliferation, and differentiation (Colognato and Yurchenco 2000). The carboxyl-terminal globular G domain of the laminin-α chain contains five sub-domains in tandem that play various biological activities. It also contains the sites recognized by several integrin receptors (Mercurio 1995, Nomizu et al. 1995, Colognato and Yurchenco 2000). Several synthetic peptides from the G domain of the laminin-α1 chain promote heparin binding, cell adhesion, neurite outgrowth, and tumor growth and metastasis.

Laminin-1, composed of α1, β1, and γ1 chains, is the most extensively characterized isoform. This protein is the earliest synthesized network-forming component during embryogenesis, and plays an important role in embryonic development and placentation (Miner et al. 1997, Colognato and Yurchenco 2000, Aumailly et al. 2000, Miner et al. 2004). Of the three chains, the α1 chain appears in epithelial basement membrane at very early stages of embryonic development, but its expression at many sites decreases during maturation (Miner et al. 1997). Laminin-2 (α2, β1, γ1) and laminin-4 (α2, β2, γ1) are the most abundant isoforms in muscle and nerve tissues (Sunada et al. 1994, Xu et al. 1994, Colognate et al. 1997). Laminin-5 (α3, β3, γ2) is an epidermis-specific isoform (Hisamatsu et al. 2003). Laminin-10 (α5, β1, γ1)/-11 (α5, β2, γ1) is widely expressed in embryonic as well as in adult tissues such as lung, heart, bone marrow, pancreas and kidney (Miner et al. 1995, Miner et al. 1997), and plays an important role during development (Miner et al. 1998,‘ Miner et al. 2004).

Several studies substantiate the relationship between anti-laminin-1 autoantibodies (autoAbs) and reproductive failure. These autoAbs were first detected in the sera of monkeys with history of reproductive failure (Carey and Klein 1989). Immunization of monkeys with mouse laminin-1 or with laminin-1 peptides caused sera embryotoxicity and infertility or spontaneous abortion (Weeks et al. 1989, Chambers et al. 1995). Passive immunization with rabbit anti-laminin-1 Abs in pregnant mice induced spontaneous abortion. We also established a mouse model that produced high titers of anti-laminin-1Abs after immunization with mouse laminin-1. Anti-laminin-1 Abs from the immunized mice caused a higher fetal resorption rate with lower embryonic and placental weights (Matalon et al. 2003). We have also demonstrated that elevated serum levels of IgG anti-laminin-1 Abs are significantly associated with recurrent first-trimester miscarriages and infertility with endometriosis (Inagaki et al. 2001, Inagaki et al. 2003). These observations suggest that anti-laminin-1 autoAbs may be responsible for reproductive failure, interfering with the early stages of pregnancy.

In the present study, we established an autoreactive IgM monoclonal anti-laminin-1 Ab (AK8) from mice immunized with mouse laminin-1 and it may develop a useful mouse model for autoimmune-mediated fetal loss. We also characterized the structure of the epitope and the specificity of this monoclonal antibody (mAb).

### Materials and methods

### *Laminins*

The following laminin isoforms were obtained from commercial sources: laminin-1 (α1, β1, γ1) derived from Engelbreth–Holm–Swarm sarcoma (EHS) (Asahi Techno Glass Co., Chiba, Japan); laminin-2 (α2, β1, γ1), laminin-10/11 [(α5, β1, γ1)/(α5, β2, γ1)] derived from human placenta (Invitrogen Co., Carlsbad, CA); and laminin-5 (α3, β3, γ2) purified from cultured human keratinocytes (Hisamatsu et al. 2003). All peptides were synthesized with Fmoc-based solid-phase strategy (Nomizu et al. 1995).

### *Establishment of monoclonal anti-laminin-1 mAb*

Balb/c mice (7 weeks old) were subcutaneously (s.c.) immunized with 20 μg of mouse laminin-1, which was isolated from Engelbreth–Holm–Swarm sarcoma (EHS) cells, with complete or incomplete Freund's adjuvant. After repeated s.c. immunizations, mice intravenously (i.v.) received a final injection of 20 μg of laminin-1. Mice were sacrificed and spleen was excised 2 days after the final injection. Spleen cells from the immunized mice were fused with mouse myeloma cells (SP2/O-Ag14), according to procedures described in Current Protocols in Immunology (Coligan et al. 1991). The fused cells were cultured in 96-well microtiter plates and the anti-laminin-1 secreting hybridomas were then cloned. Hybridomas that produced AK8 mAbs were obtained using serum-free cultured medium (CD Hybridoma Medium, (1X) liquid, GIBCO™, Invitrogen Co., Carlsbad, CA).

### *Enzyme-linked immunosorbent assay (ELISA)*

ELISA for anti-laminin-1 antibodies was performed as described by Inagaki et al. (2001). Briefly, polystyrene plates (Immulon 1B, Thermo Electron, San Jose, CA) were coated with the purified laminin-1 (10 μg/ml, 50 μl/well) by overnight incubation at 4°C. Plates were blocked with 10% fetal bovine serum. Diluted samples (100 μl/well) were applied to microwells and incubated for 1 h at room temperature. Horseradish peroxidase-labeled anti-mouse IgG or IgM Abs were incubated for 1 h. *O*-Phenylenediamine solution containing $H_2O_2$ was reacted for 10 min, and the reaction terminated with 2N $H_2SO_4$. Optical density (OD) was measured at 490 nm.

*Western blot analysis*

SDS-polyacryamide gel (7%) electrophoresis (SDS-PAGE) was performed under the reduced condition according to the method of Laemmli, (1970). Following electrophoresis, the proteins were transferred to a nitrocellulose membrane, blocked for 30 min with PBS containing 5% skim milk (PBS–SM), and then incubated overnight at 4°C with purified AK8 mAb. After washing, alkaline phosphatase-conjugated anti-mouse IgM Abs in PBS–SM were incubated for 1 h. After the extensive washing, color was developed with nitroblue tetrazolium-5-bromo-4-chloro-3-indolylphosphate (BCIP).

*Phage displayed random peptide library*

To predict the epitope recognized by the AK8 mAb a phage displayed random peptide procedure was performed with a Ph.D.™-12 Phage Display Peptide Library Kit (New England Biolabs, Inc., Tozer Road Beverly, MA) following the guidelines of the kit's technical manual.

*Modeling the epitope structure recognized by AK8 mAb*

The possible tertiary structure of the epitope recognized by AK8 mAb was predicted by the flexible alignment method implemented in the MOE commercial software package (Chemical Computing Group Inc.; http://www.chemcomp.com/). We used the conformations of synthesized peptides (12 mer) located in the G domain of the laminin-α1 that reacted with the mAb and the consensus sequences obtained by epitope mapping with the phage displayed random peptide library. The objective was to find similar positions of functional groups among a number of peptides with the stochastic mode by 200 iterations of the flexible alignments method. Energy minimization was performed with a MMFF94× force field, 10 Å cut-off distance of non-bonded interaction and 200 steps limitation for each minimization. The similarities of hydrogen bond donor/acceptor, aromaticity, hydrophobicity, partial charge and volume of each functional group were taken into consideration with superimpositions of these peptides with a 500 configuration limit. This method was originally applied for small molecule fittings, but it could be employed to study correct fittings of peptide structures.

*Immunohistochemical staining*

Mouse uterine tissues obtained at day 7.5 of gestation and human endometrial tissues were placed in optimal cutting temperature Tissue-Tek OCT compound (Sakura Finetschnical Co. Ltd., Tokyo, Japan), and frozen in liquid nitrogen for immunohistochemical staining. Serially cut cryostat sections (~6 μm thick) were picked up on separate slides, air-dried, fixed in 100% acetone at 4°C for 10 min and washed with PBS. The slides were incubated with 0.3% hydrogen peroxidase to quench endogenous peroxidase activity. The sections were then incubated with AK8 mAb for 60 min at 37°C, followed by sequential incubations with biotinylated anti-mouse IgM Abs and horseradish peroxidase-labeled streptavidin. The staining was completed with substrate-chromogen solution, dimethylaminobendizine (DAB). The specimens were examined by Olympus BX50 microscope equipped with appropriate filters.

### Results

*Establishment of hybridoma secreting mAb against laminin-1*

One stable hybridoma cell line secreting anti-laminin-1 Ab, named AK8, was only established after ten independent cell fusions of splenocytes from mouse laminin-1 immunized mice with mouse myeloma cells (SP2/0-Ag14). The isotype of AK8 mAb was identified to be IgM (μ, κ). Hybridoma cells were maintained in serum free medium (CD Hybridoma Medium, GIBCO™). Cell line cultured supernatant was collected, and IgM enriched fractions were prepared from the supernatant.

*Specificity of AK8 mAb*

ELISA was first performed on microtiter plates coated with various isoforms of laminin-1 to characterize the specificity of AK8 mAb, as previously described (Inagaki et al. 2001). AK8 mAb only bound to mouse laminin-1 (α1, β1, γ1), but not to other human laminin isoforms, i.e. laminin-2 (α2, β1, γ1), laminin-5 (α3, β3, γ2), or laminin-10/11 [(α5, β1, γ1) and (α5, β2, γ1), respectively] (**Figure** 1). The specificity was confirmed by Western blot analysis. Mouse laminin-α1 chain was only stained with AK8 mAb but neither β1 nor γ1 chains were stained (**Figure** 2). As shown in **Figure** 3, AK8 mAb bound to both the whole molecule of mouse laminin-1 and its G domain on a solid phase. Also, the AK8 mAb binding to laminin-1 was inhibited in a dose-dependent fashion by addition of exogenous G domain. Thus, we concluded that the epitope for AK8 is located in the G domain of laminin-αl chain.

*A possible tertiary structure of AK8's epitope*

To predict the fine specificity of AK8 mAb, we performed ELISA using G domain overlapped synthesized peptides. Significant high binding was observed with nine synthesized peptides on a solid

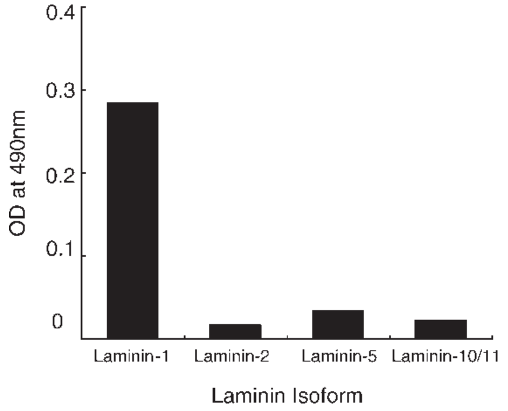

Figure 1. Reactivity of AK8 mAb to solid phase laminin isoforms. Laminin-1 (α1, β1, γ1; from mouse EHS); laminin-2 (α2, β1, γ1; from human placenta); laminin-5 (α3, β3, γ2; from cultured human keratinocytes); laminin-10/11 [(α5, β1, γ1)/(α5, β2, γ1); from human placenta]. ELISA was performed as described in "Materials and methods" section.

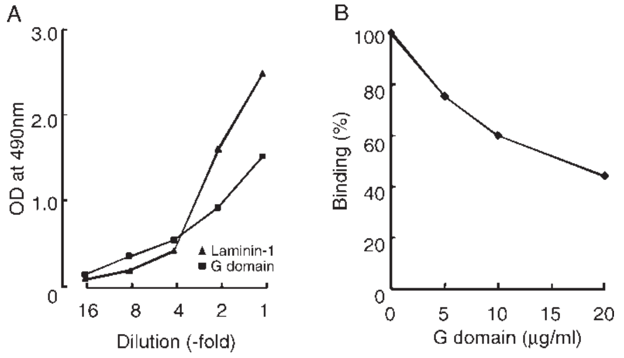

Figure 3. Binding profile of AK8 mAb to the whole molecule and to the G domain of laminin-1. A: Direct binding of AK8 mAb to solid phase laminin-1 and its G domain; B: Inhibition of exogenous G domain on AK8 binding to laminin-1. ELISA was performed as described in "Materials and methods" section.

phase, but we could not find any homology in their primary amino acid sequences. Actually, a model was constructed with eight out of nine peptide sequences (Table I) recognized by AK8 mAb. The AG73 segment was the only one excluded due to reduced similarity. Two of these peptides (in bold in Table I) did show bioactivity. In addition, Table I lists three different consensus peptide (12 mer) sequences obtained by phage displayed random peptide library.

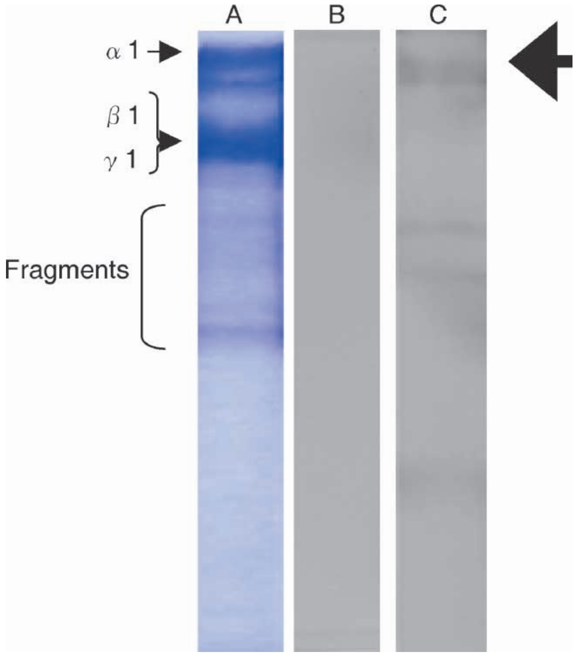

Figure 2. Western blot analysis on AK8 mAb. laminin-1 (from mouse EHS cells) was run on 7% SDS-PAGE gel under the reduced condition. Lane A: staining with Coomassie Brilliant Blue; lane B (Western blot): control; lane C (Western blot): with AK8 mAb. Molecular weight of the whole molecule, α1 chain, β1 chain, and γ1 chain of laminin-1 are 900, 440, 220, and 205 kDa, respectively.

None of the twelve peptides showed high homology in their amino acid sequence. We tried to predict the common tertiary structure of the AK8 epitope by the flexible alignment method. Superimposed conformations of the eight G domain synthesized peptides and three phage library peptides are shown in Figure 4(A) and (B), respectively. Both conformations have five common regions, as follows: (i) positive charged group; (ii) hydrophilic group; (iii) wide hydrophobic region; (iv) hydrophilic group; and (v) aromatic ring. These regions were aligned in a similar direction in both peptide groups. It was assumed that some regions with these positional relationships would be a requisite for constructing the epitope.

### *Immunohistochemical localization of laminin-1*

Immunoreactivity of AK8 mAb was selectively found in the basement membranes of endometrial epithelium in pregnant mouse at day 7.5 of gestation (Figure 5). Basement membranes of glandular epithelium of human endometrium were also significantly stained with AK 8 mAb (Figure 6). There was 76.2, 85.9, and 85.7% homology between mouse and human laminin α1, β1, and γ1 chains, respectively

Table I. Peptide sequences recognized by AK8.

| Overlapping peptide in the G domain | Consensus peptides sequence from phage displayed random peptide library |
|---|---|
| AG16: LMFVGGLGGQIK | 1: TTRSPTFPTYTY |
| **AG22: SSFHFDGSGYAM** | 2: SNRELASTHPAS |
| AG25: VILFSTFSPNGL | 3: AGTPLPTFGMTD |
| AG26: PNGLLFYLASNG | |
| AG47: FATKNSSGILLV | |
| AG63: IKNVVLDAQLLD | |
| **AG76: QNQMDYATLQLQ** | |
| AG97: SAKVDAIGLEIV | |

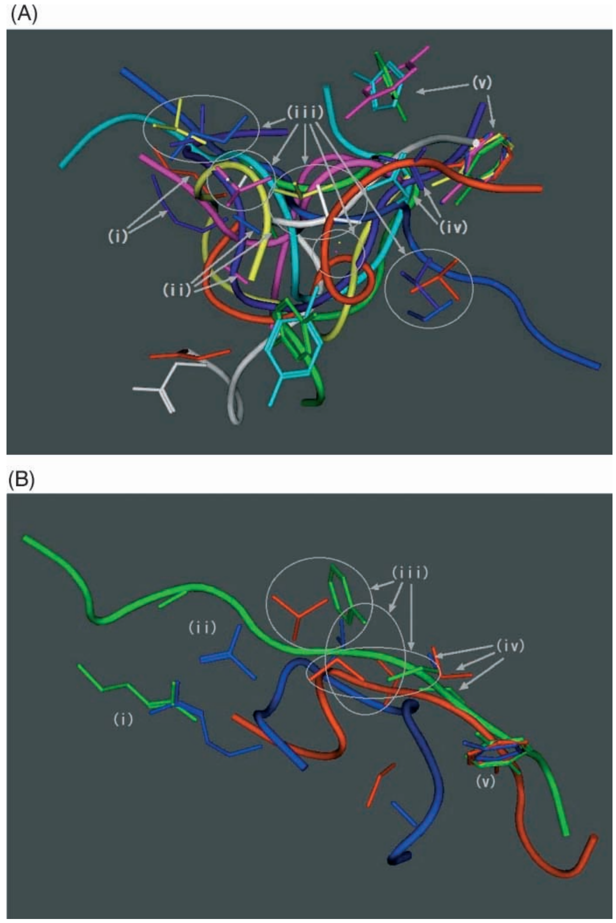

Figure 4. Possible conformations from the flexible alignment analysis of an epitope for AK8 mAb from (A) sequences of eight AK8 mAb-reactive overlapping synthesized peptides in the G-domain, and (B) consensus peptide sequences obtained by the epitope mapping with phage displayed random peptide library. Each molecular model was shown with Cα trace and side chains related with superimpositions of similar functional groups among these peptides. In panel A, the synthesized peptides, AG16, AG22, AG25, AG26, AG47, AG63, AG76, and AG97, were colored by red, green, blue, violet red, yellow, white, light blue, and violet blue. Superimpositions were resulted as follows; (i) positive charged group: $K^{12}$ (AG16)/$K^{3}$ (AG97), (ii) hydrophilic group: $S^{2}$ (AG22)/$S^{5}$ (AG25)/$S^{10}$ (AG26), (iii) wide hydrophobic region: $I^{2}$ (AG25)/$L^{10}$ (AG47)/$V^{4}$ (AG97), $L^{8}$ (AG26)/$V^{5}$ (AG63), $L^{11}$ (AG47)/$V^{4}$ (AG63), $A^{9}$ (AG26)/$A^{2}$ (AG47)/$A^{8}$ (AG76), and $L^{7}$ (AG16)/$P^{9}$ (AG25)/$L^{9}$ (AG97), (iv) hydrophilic group: $Q^{4}$ (AG76)/$E^{10}$ (AG97), (v) two regions (or the diversity of one region) of aromatic group: $F^{3}$ (AG16)/$F^{5}$ (AG22)/$F^{7}$ (AG25)/$F^{1}$ (AG47) and $H^{4}$ (AG22)/$Y^{7}$ (AG26)/$H^{1}$ (AG76). Other superimpositions similar to (iv) and (v) were also shown in $Q^{10}$ (AG16)/$Q^{9}$ (AG63) and $Y^{10}$ (AG22)/$Y^{7}$ (AG76). In panel B, peptide, #1, #2, and #3 were colored by red, green, and blue. Superimpositions were resulted as follows: (i) positive charged group of $R^{3}$ (#2)/$R^{3}$ (#3), (ii) hydrophilic group: $S^{4}$ (#2)/$N^{2}$ (#3), (iii) wide hydrophobic region: $L^{5}$ (#1)/$F^{7}$ (#2), $P^{6}$ (#1)/$L^{5}$ (#3), and $P^{4}$ (#1)/$P^{8}$ (#2), (iv) hydrophilic group: $T^{7}$ (#1)/$T^{9}$ (#2)/$S^{7}$ (#3), (v) aromatic group: $F^{8}$ (#1)/$Y^{10}$ (#2)/$H^{8}$ (#3). The other superimposition of $T^{3}$ (#1)/$S^{12}$ (#3) was shown as similar to (iv).

(GENETYX-Mac 10.1). Thus, AK8 may cross-react with human α1 chain located in the basement membranes of glandular epithelium of human endometrium.

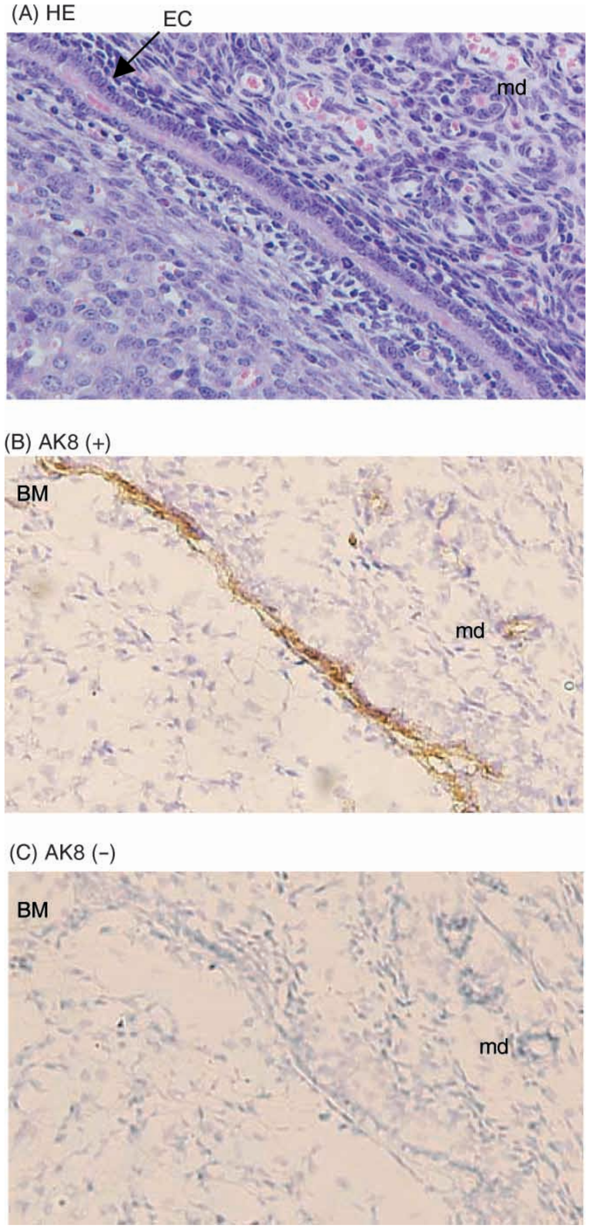

Figure 5. Immunostaining of endometrium in pregnant mouse at day 7.5 of gestation with AK8 mAb (magnification × 100). Hematoxylin eosin (HE) staining (A); immunostaining (B) with and (C) without AK8 mAb. EC: endometrial cell; BM: basement membrane of endometrial epithelium and md: maternal decidua.

### Discussion

To date, several mouse mAbs against human laminin isoforms have been produced and used to study the histological distribution of laminins. However, no murine mAbs have been produced by immunizing mice with mouse-derived laminin-1 (purified from EHS sarcoma cell). In the present study, IgG and IgM titers were frequently elevated in mice during the immunization period, and cell fusion processes were successfully performed routinely in our laboratory, but only a small number of hybridomas was obtained 1 week after the cell

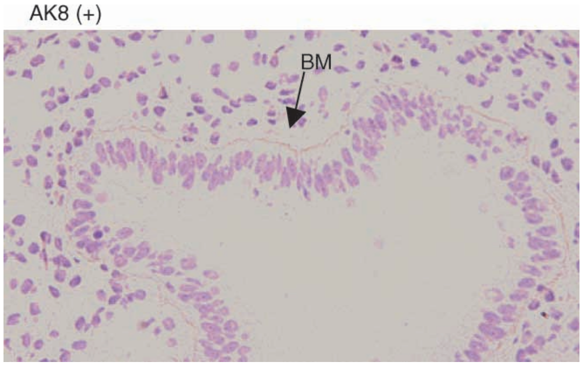

Figure 6. Immunostaining of human endometrium with AK8 mAb (magnification × 200). BM: basement membrane of glandular epithelium.

fusion. After ten cell fusion trials with our routine procedure, only one stable hybridoma cell line secreting an IgM mAb (AK8) was established. The AK8 hybridoma cell line was continuously maintained in MEM with 10% fetal bovine serum and/or the serum free medium (CD Hybridoma Medium, (1 ×) liquid, GIBCO™). This may be the first report of a hybridoma secreting an autoreactive mAb against mouse laminin-1, such as AK8 mAb.

We recently reported that anti-laminin-1 Abs induced by active immunization with mouse laminin-1 from EHS cells caused a higher fetal resorption rate with lower embryonic and placental weights in the immunized mice as compared with controls. Rasmussen et al. (1994) reported that laminin mAbs affect the development of cultured rat embryos. Shim et al. (1997) clarified that the expression of laminins is increased at sites of cell proliferation and differentiation along with implantation process by using ovarian steroids. Thus, anti-laminin-1 Abs may affect the implantation and/or the fetal development in the mice model. Our hypothesis is that such autoAbs may directly affect the development and differentiation of basement membranes in placenta and/or embryos through the inhibition of laminin polymerization in conjunction with cell anchorage. Furthermore, passive immunization models may be developed using AK8 mAb if IgM Ab could be diffused to basement membranes of glandular epithelium of endometrium during the peri-implantation period and/or diffuse to basement membranes of embryos through placenta. The model will be important to demonstrate that anti-laminin-1 Abs is pathogenic.

Interestingly, AK8 mAb stained the basement membranes of glandular epithelium of mouse and human endometrium (Figures 5 and 6). We routinely detected human IgM and/or IgG anti-laminin-1 autoAbs by using mouse laminin-1 from EHS cells as antigen in solid phase ELISA. The cross-reaction of AK8 with human laminin-1 may be possible because homology of amino acid sequence of human and mouse laminin-1 is around 80% (by GENETYX).

We performed direct and inhibition ELISA to clarify specificity of AK8. The results showed that AK8 is specific to the laminin-α1 chain G domain. Furthermore, to characterize the fine structure of the epitope recognized by AK8 mAb, we performed two epitope mappings: one by ELISA using overlapping synthesized peptides in the laminin-α1 G domain and another by phage displayed random peptide library. Unfortunately, we did not get any homology in the primary peptide sequences in either system. Then, we tried to predict a tertiary structure of epitope for AK8 mAb with eight and three non-homologous peptide sequences from these two individual systems, and a particular common structure was successfully constructed. However, the structure constructed by this modeling should be confirmed by other procedures.

Because there is a limitation of placenta permeability to protect fetuses, IgM may not reach the fetus during pregnancy. Yet, Hjortberg et al. (1991) demonstrated that when purified immunoglobulins, both non-specific mouse IgG and IgM myeloma and specific IgG and IgM anti-blastocyst, were injected i.v. they were able to enter the uterine cavity of the mouse. It was further shown that Abs against morulae and/or blastocysts may be able to influence the development of the embryo during pre and peri-implantation periods, and could serve as a useful model for experiments related not only to infertility, but also to the identification of immunological contraceptive procedures.

In conclusion, we have developed a mouse IgM autoreactive mAb, AK8, specific to mouse laminin-1. The mAb recognized a particular structure of the G domain of the laminin-α1 chain and cross-reacted with human laminin-1. Anti-laminin-1 autoAbs have been demonstrated to be present in sera of patients with recurrent abortions. AK8 mAb may be useful for development of a mouse model for autoAb-mediated fetal loss by active immunization.

### References

Aumailley M, Pesch M, Tunggal L, Gaill F, Fassler R. 2000. Altered synthesis of laminin 1 and absence of basement membrane component deposition in β1 integrin-deficient embryoid bodies. J Cell Sci 113:259–268.

Carey SW, Klein NW. 1989. Autoantibodies to laminin and other basement membrane proteins in sera from monkeys with histories of reproductive failure identified by cultures of whole rat embryos. Fertil Steril 51:711–718.

Chambers BJ, Klein NW, Conrad SH, Ruppenthal GC, Sackett GP, Weeks BS, Kleinman HK. 1995. Reproduction and sera embryotoxicity after immunization of monkeys with the laminin peptides YIGSR, RGD, and IKVAV. Proc Natl Acad Sci USA 92:6818–6822.

Coligan JE, Kruisbeek AM, Margulies DH, Shevach EM, Stober W. 1991. Currnet Protocols in Immunology. NY: 2.0.1-2.11.8

Greene Publishing Associates and Wiley-Interscience, John Wiley and Sons.

Colognato H, Yurchenco PD. 2000. Form and function: The laminin family of heterotrimers. Dev Dyn 218:213–234.

Colognato H, MacCarrick M, O'Rear JJ, Yurchenco PD. 1997. The laminin α2-chain short arm mediates cell adhesion through both the α1β1 and α2β1 integrins. J Biol Chem 272:29330–29336.

Hisamatsu Y, Nishiyama T, Amano S, Matsui C, Ghohestani R, Hashimoto T. 2003. Usefulness of immunoblotting using purified laminin 5 in the diagnosis of anti-laminin 5 cicatricial pemphigoid. J Dermatol Sci 33:113–119.

Hjortberg M, Jin M, Larsson A, Nilsson BO. 1991. Immunoglobulins and monoclonal anti-blastocyst antibodies in the mouse uterine secretion during the early pre-implantation period. J Reprod Immunol 20:277–287.

Inagaki J, Matsuura E, Nomizu M, Sugiura-Ogasawara M, Katano K, Kaihara K, Kobayashi K, Yasuda T, Aoki K. 2001. IgG anti-laminin-1 autoantibody and recurrent miscarriages. Am J Reprod Immunol 45:232–238.

Inagaki J, Sugiura-Ogasawara M, Nomizu M, Nakatsuka M, Ikuta K, Suzuki N, Kaihara K, Kobayashi K, Yasuda T, Shoenfeld Y, Aoki K, Matsuura E. 2003. An association of IgG anti-laminin-1 autoantibodies with endometriosis in infertile patients. Hum Reprod 18:544–549.

Laemmli UK. 1970. Cleavage of structural proteins during the assembly of the head of bacteriophage T4. Nature 227:680–685.

Li S, Harrison D, Carbonetto S, Fassler R, Smyth N, Edgar D, Yurchenco PD. 2002. Matrix assembly, regulation, and survival functions of laminin and its receptors in embryonic stem cell differentiation. J Cell Biol 157:1279–1290.

Matalon ST, Blank M, Matsuura E, Inagaki J, Nomizu M, Levi Y, Koike T, Shere Y, Ornoy A, Shoenfeld Y. 2003. Immunization of naive mice with mouse laminin-1 affected pregnancy outcome in a mouse model. Am J Reprod Immunol 50:159–165.

Mercurio AM. 1995. Laminin receptors: Achieving specificity through cooperation. Trends Cell Biol 5:419–423.

Miner JH, Lewis RM, Sanes JR. 1995. Molecular cloning of a novel laminin chain, α5, and widespread expression in adult mouse tissues. J Biol Chem 270:28523–28526.

Miner JH, Patton BL, Lentz SI, Gilbert DJ, Snider WD, Jenkins NA, Copeland NG, Sanes JR. 1997. The laminin α chains: Expression, developmental transitions, and chromosomal locations of α-5, identification of heterotrimeric laminins 8–11, and cloning of a novel α3 isoform. J Cell Biol 137:685–701.

Miner JH, Cunningham J, Sanes JR. 1998. Roles for laminin in embryogenesis: exencephaly, syndactyly, and placentopathy in mice lacking the laminin α5 chain. J Cell Biol 143:1713–1723.

Miner JH, Li C, Mudd JL, Go G, Sutherland AE. 2004. Compositional and structural requirements for laminin and basement membranes during mouse embryo implantation and gastrulation. Development 131:2247–2256.

Nomizu M, Kim WH, Yamamura K, Utani A, Song SY, Otaka A, Roller PP, Kleinman HK, Yamada Y. 1995. Identification of cell binding sites in the laminin ( 1 chain carboxyl-terminal globular domain by systematic screening of synthetic peptides. J Biol Chem 270:20583–20590.

Rasmussen MV, Klein NW, Abrahamson DR, Chung AE. 1994. Effects of laminin monoclonal antibodies on the development of cultured rat embryos. Teratology 49:20–28.

Shim C, Choi D, Kwon HB, Kim K. 1997. Expression of laminin chain-specific gene transcripts in mouse uterine tissues during peri-implantation period. Mol Reprod Dev 48:176–184.

Sunada Y, Bernier SM, Kozak CA, Yamada Y, Campbell KP. 1994. Deficiency of merosin in dystrophic dy mice and genetic linkage of laminin M chain gene to dy locus. J Biol Chem 13(269):13729–13732.

Tunggal P, Smyth N, Paulsson M, Ott MC. 2000. Laminins: Structure and genetic regulation. Microsc Res Tech 51:214–227.

Weeks BS, Klein NW, Kleinman H, Frederickson T, Sackett GP. 1989. Laminin immunized monkeys develop sera toxic to cultured rat embryos and fail to reproduce. Teratology 40:47–57.

Xu H, Christmas P, Wu XR, Wewer UM, Engvall E. 1994. Defective muscle basement membrane and lack of M-laminin in the dystrophic dy/dy mouse. Proc Natl Acad Sci USA 91:5572–5576.

